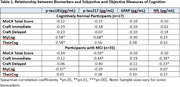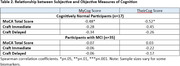# Greater subjective cognitive decline is associated with higher plasma Alzheimer’s disease biomarkers in cognitively normal Hispanics living in Miami, FL

**DOI:** 10.1002/alz.086425

**Published:** 2025-01-09

**Authors:** Warren W Barker, Yaimara Gonzalez Pinero, Karen Velasquez, Joanna Gonzalez, Idaly Velez‐Uribe, Mileidys Herrera, Ranjan Duara, David P. Salmon, Douglas R. Galasko, Maria J. Marquine, Zvinka Zlatar

**Affiliations:** ^1^ Mount Sinai Medical Center, Miami Beach, FL USA; ^2^ University of California, San Diego, La Jolla, CA USA; ^3^ Duke University, Durham, NC USA

## Abstract

**Background:**

Subjective cognitive decline (SCD), or a person’s perception of changes in their cognitive abilities, has been identified as a possible early marker of preclinical Alzheimer’s disease (AD) in non‐Hispanic Whites; however, research is lacking about the clinical utility of SCD in diverse populations. This study investigated the associations of self and informant reports of SCD, plasma biomarker profiles of AD, and objective cognitive performance in Hispanic older adults living in Miami.

**Method:**

Hispanic participants enrolled in the 1Florida Alzheimer’s Disease Research Center who completed neuropsychological testing and blood draws for biomarker analysis were eligible. Plasma biomarkers included phosphorylated tau (p‐tau217 and p‐tau181), glial fibrillary acidic protein (GFAP), and neurofilament light chain (NfL). The Subjective Cognitive Decline Questionnaire self (MyCog) and informant (TheirCog) versions were used to assess SCD. Cognitive tests included the Montreal Cognitive Assessment (MoCA) and the Craft story 21 verbatim immediate (Craft‐Im) and delayed recall (Craft‐Del). Relationships between plasma biomarkers, cognitive performance, and SCD were explored using Spearman's rank correlation, with an uncorrected p‐value of 0.05.

**Result:**

The cohort included 52 Hispanics (age 73.3±7.3 years; education 15.2±3.0 years; 63% female; 96% from Cuba or South America) with a consensus diagnosis of cognitively normal (CN, n=17) or mild cognitive impairment (MCI, n=35). Among CN participants (Tables 1 and 2), greater MyCog scores were significantly associated with higher p‐tau217 (r_s_=0.68), p‐tau181 (r_s_=0.58) and lower MoCA scores (r_s_=‐0.48). Greater TheirCog scores were significantly associated with higher p‐tau181 (r_s_=0.58) and lower MoCA scores (r_s_=‐0.52). Among MCI participants (Tables 1 and 2), greater MyCog scores were associated with lower p‐tau181 (r_s_=‐0.37). There were significant correlations between various cognitive test scores and p‐tau217 (MoCA, r_s_=‐0.50; CraftIm, r_s_=‐0.44) and NfL (CraftIm, r_s_=‐0.34; CraftDel, r_s_=‐0.33).

**Conclusion:**

Greater self and informant reported SCD are related to higher AD plasma biomarker load and worse cognition in a small sample of CN Hispanics, suggesting potential clinical utility. Self‐reported SCD was inversely related to plasma biomarkers in those with MCI, suggesting loss of insight. Larger and more diverse longitudinal studies are needed to elucidate if CN Hispanics with elevated SCD and AD plasma biomarkers progress to MCI and AD.